# Heterogeneity of insulin resistance and beta cell dysfunction in gestational diabetes mellitus: a prospective cohort study of perinatal outcomes

**DOI:** 10.1186/s12967-018-1666-5

**Published:** 2018-10-24

**Authors:** Yingfeng Liu, Wolin Hou, Xiyan Meng, Weijing Zhao, Jiemin Pan, Junling Tang, Yajuan Huang, Minfang Tao, Fang Liu

**Affiliations:** 1 Shanghai Key Laboratory of Diabetes, Department of Endocrinology & Metabolism, Shanghai Jiao-Tong University Affiliated Sixth People’s Hospital, Shanghai Clinical Medical Center of Diabetes, Shanghai Key Clinical Center of Metabolic Diseases, Shanghai Institute for Diabetes, 600 Yishan Road, Shanghai, 200233 China; 2 Department of Obstetrics and Gynecology, Shanghai Clinical Center for Severe Maternal Rescue, Shanghai Jiao-Tong University Affiliated Sixth People’s Hospital, Shanghai, China

**Keywords:** Gestational diabetes mellitus, Insulin resistance, Beta cell dysfunction, Perinatal outcome

## Abstract

**Background:**

Insulin resistance and beta cell dysfunction were reported to be responsible for gestational diabetes mellitus (GDM). However, little is known about the heterogeneity of these factors and its influences on perinatal outcomes. We investigated whether subtypes of insulin resistance and beta cell dysfunction in gestational diabetes mellitus have different impacts on perinatal outcomes.

**Methods:**

In this prospective cohort study, we followed 554 pregnant women and glucose challenge test was performed at 24–28th weeks of their gestation. Women with plasma glucose ≥ 7.8 mmol/L would be included and advised to undergo the diagnostic 75-g, 3-h oral glucose tolerance test. According to indices of measuring insulin resistance or beta cell function were below the 25th percentile of women with normal glucose tolerance (NGT), women with GDM were defined as three subtypes: GDM with the beta cell dysfunction, GDM with the insulin resistance defect or GDM with both traits mentioned above (GDM-mixed). Perinatal outcomes were documented.

**Results:**

The levels of prepregnancy and maternal BMI in the GDM-mix group were higher compared to women in the NGT group (23.2 ± 4.0 vs 20.8 ± 3.7 kg/m^2^, *P *< 0.001; 24.5 ± 4.3 vs 21.8 ± 3.4 kg/m^2^, *P *< 0.001, respectively). Furthermore, women in GDM-mix group more likely to be subjected to LGA (*P *= 0.008) adverse perinatal outcomes (*P *= 0.005), although these differences were normalized after adjusting age, prepregnancy and maternal BMI (GDM-mix vs. NGT: *P *= 0.141 for LGA and *P *= 0.186 for adverse outcomes). On the other hand, all perinatal outcomes were similar between other two GDM subgroups and NGT group.

**Conclusions:**

Women with GDM display respective characteristics on metabolism disorders and confer discriminating risks of adverse perinatal outcomes because of this heterogeneity.

**Electronic supplementary material:**

The online version of this article (10.1186/s12967-018-1666-5) contains supplementary material, which is available to authorized users.

## Background

Gestational diabetes mellitus (GDM), a glucose intolerance with onset or first diagnosed during pregnancy [1], makes a tremendous contribution to adverse perinatal outcomes to both the mother and developing fetus [2]: Common maternal outcomes of GDM include the incremental risk of caesarean section, preeclampsia or proteinuria and likelihood of developing type 2 diabetes (T2D) in the future; for infants, fetal complications, connected with GDM, comprise neonatal hypoglycemia, stillbirth and macrosomia or large for gestational age (LGA), sometimes birth trauma, shoulder dystocia especially, as well [3–5].

Although exact mechanisms responsible for the development of hyperglycemia during pregnancy are not fully understood, insulin resistance has been identified as the catalyst of GDM with advancing gestation. Chronic inflammation factors, such as tumor necrosis factor α, and placental-derived hormones, such as placental lactogen and growth hormone, have been verified to be contributors to this increasing level of insulin resistance [6, 7] and when women fail to adapt to these physiological changes, they develop the glucose intolerance during pregnancy. In addition, the majority of women who develop GDM also experience a significant impairment in beta cell function which leads to initial postprandial and later fasting hyperglycemia [8, 9], and an elevated ratio of proinsulin to insulin was also observed in women with GDM to indicate beta cell dysfunction [10].

It has been suggested that GDM, the same as T2D, is a heterogeneous disease [11] and most studies focused on diverse subtypes of maternal body mass index (BMI), weight gain or level of glucose [12–16]. However, few studies characterized GDM based on insulin resistance and beta cell dysfunction, which are linked up with the BMI and circulating glucose concentrations [17], important predictors of perinatal outcomes. Therefore, we carried out a prospective cohort study to clarify the heterogeneous impact of insulin resistance and beta cell dysfunction in GDM on perinatal outcomes.

## Methods

### Study population and design

A prospective cohort study was conducted and all subjects were recruited in a prospective cohort of pregnant women from January 2015 to June 2016 at the Department of Obstetrics and Gynecology of Shanghai Jiao-Tong University Affiliated Sixth People’s Hospital. Their clinical and biochemical profiles longitudinally were collected from initial visit to delivery. In order to include as many as pregnant women with GDM, 50-g 1-h oral glucose tolerance was firstly performed among them at 24–28th weeks of gestation. Women with plasma glucose  ≥ 7.8 mmol/L were included and advised to undergo the diagnostic 75-g, 3-h oral glucose tolerance test (OGTT), and GDM was diagnosed according to the International Association of Diabetes and Pregnancy Study Group (IADPSG) [18]. Women with the history of diabetes and multiple gestations were excluded. Five hundred and fifty-five women in total were enrolled at the baseline. We excluded 1 woman with the history of diabetes, 16 women with preeclampsia, 12 women because of multiple gestations, 70 women because of missing data on covariates. Finally, 456 subjects were eligible and their clinical data were available in the study. This study was approved and supervised by the Ethics Committee of the Shanghai Jiao-Tong University Affiliated Sixth People’s Hospital. All the participants signed the informed consents. The study was administered according to the Declaration of Helsinki.

To outline the heterogeneity of GDM, we reckoned the distributions of insulin sensitivity and secretion in women with normal glucose tolerance (NGT) as the reference. We considered women with GDM to have an insulin resistance or beta cell dysfunction if indices of measuring insulin resistance or beta cell function were below the 25th percentile, respectively. Women with GDM were classified into four subtypes based on the characteristic present: GDM with a predominant beta cell dysfunction (GDM-dysfunction), GDM with a predominant insulin resistance defect (GDM-resistance), or GDM with both traits mentioned above (GDM-mixed). If one participant with GDM had indices of both insulin sensitivity and beta cell function above the 25th percentile, she was not included from subgroup analyses (Table 1 and Additional file 1: Table S1 listed clinical information and perinatal outcomes of women in this group—GDM-normal).

**Table 1:** Baseline characteristics of pregnant women in different GDM subgroups

	GDM-resistance	*P* value	GDM-dysfunction	*P* value	GDM-mix	*P* value	GDM-normal	*P* value	All GDM	*P* value	NGT
Participants (n)	35 (17.0% of GDM)		43 (20.9% of GDM)		79 (38.3% of GDM)		49 (23.8% of GDM)		206		250
Age (years)	31.0 (7.5)	> 0.999	31.0 (6.0)	0.021	32.0 (6.0)	< 0.001	31.0 (4.0)	0.824	32.0 (5.0)	< 0.001	29.0 (5.0)
Prepregnancy BMI (kg/m^2^)	21.5 (3.2)	0.231	21.6 (3.2)	0.405	23.2 (4.0)	< 0.001	20.7 (3.3)	> 0.999	22.0 (3.7)	< 0.001	20.8 (3.7)
Maternal BMI (kg/m^2^)	23.1 (4.0)	0.071	22.2 (2.9)	0.45	24.5 (4.3)	< 0.001	21.3 (3.3)	> 0.999	23.2 (3.7)	< 0.001	21.8 (3.4)
Gestational age at weight measuring (weeks)	13.3 (3.5)	–	13.7 (3.3)	–	13.9 (2.9)	–	13.9 (2.4)	–	13.7 (2.8)	0.073	14.0 (3.6)
Maternal BMI change (kg/m^2^)	0.8 (0.9)	–	1.1 (1.1)	–	0.8 (1.3)	–	0.8 (0.8)	–	0.8 (1.0)	0.969	0.8 (1.1)
ALT (U/L)	13 (7.5)	–	14.5 (13.0)	–	13 (10.0)	–	12 (8.5)	–	13 (10.8)	0.753	13 (11.0)
History of hypertension (n)	0	–	0	–	4 (5.1)	–	2 (4.1)	–	6 (2.9)	0.007	0
Smoking before or during pregnancy (n)	0	–	0	–	0	–	0	–	0	–	0
Nulliparous (n)	25 (71.4)	–	27 (62.8)	–	53 (67.1)	–	32 (65.3)	–	137 (66.5)	0.173	181 (72.4)

Data are median (IQR) for continuous variables and n (%) for categorical variables

Differences between GDM subgroups and women with NGT were evaluated with the Kruskal–Wallis test for continuous variables and χ^2^ or Fisher exact test for categorical variables

When the *P* value was significant (< 0.05), Dunn’s multiple comparisons test or χ^2^ or Fisher exact test was performed to evaluate the difference between each GDM subtype and NGT group. *P* values for pairwise comparisons were adjusted by Bonferroni correction

### Data collection and laboratory measurements

All the participants completed a questionnaire with the following data: general information about the present and previous illness, reproductive history, medication, alcohol consumption and smoking status. Height and weight were measured by the same physician during the health check-up. BMI was calculated as body weight (in kg)/height (in m^2^). The level of serum alanine aminotransferase (ALT) was measured in 13th–16th gestation week and determined by an automatic analyzer (7600-020 biochemistry automatic analyzer, Hitachi, Tokyo, Japan) and the normal range was 0–65 U/L. Plasma glucose and insulin values at 0, 30, 60, 120, and 180 min were obtained from the diagnostic 75-g, 3-h OGTT and levels of plasma glucose were determined by glucose oxidase method, the linearity range was 0–35 mmol/L. HbA1c and glycated serum albumin (GA) were measured at 24–28th weeks of gestation and determined by high-pressure liquid chromatography and by the liquid enzymatic assay, respectively. Insulin concentrations were determined with a 2-site chemiluminescent enzyme immunometric assay for the immulite automated analyzer (Diagnostic Products, Los Angeles, CA), the linearity range was 0.02–1000 μU/mL. HOMA2-IR at http://www.dtu.ox.ac.uk (accessed on 11 January 2016). Insulinogenic indices (a measure of insulin release) were calculated as the ratio between Δ (0–30 min) for insulin and glucose during OGTT [19]. Insulin sensitivity was assessed using the Matsuda index $[ 10,000/\surd ({\text{fasting glucose}} \times {\text{fasting insulin}}) \times ({\text{mean glucose}}_{{0{-} 1 80}} \times {\text{mean insulin}}_{{0{-} 1 80}} )]$ [20]. Beta cell function was measured by disposition index (insulinogenic index/HOMA2-IR).

### Perinatal outcomes

Gestational week at delivery, mode of delivery, birth weights, gender of offspring and Apgar score were obtained from the medical record. Fetal growth was assessed in 1 week before the delivery by a trained technician, and measurements were obtained using a ultrasonography (Philips iE33 ultrasound system and a 5-MHz probe). Biparietal diameter (BPD), femur length (FL) and amniotic fluid index (AFI) were used to evaluate the fetal growth. Adverse outcomes are including: LGA, neonatal hypoglycemia and cesarean delivery. LGA was defined as birth weight ≥ 90th percentile for completed week of gestational age based on the sex-specific Ref. [21] and birth weight z value was calculated also by this reference. Neonatal hypoglycemia was diagnosed according to the criterion that the blood glucose concentration of is less than 2.6 mmol/L that McKinlay et al. [22] claimed.

### Statistical methods

Data were expressed as median ± interquartile range (IQR) for continuous variables and percentages (%) for categorical variables. Differences between GDM subgroups and women with NGT were evaluated with the Kruskal–Wallis test for continuous variables and χ^2^ or Fisher exact test for categorical variables. When the *P* value was significant (< 0.05), Dunn’s multiple comparisons test or χ^2^ or Fisher exact test was performed to evaluate the difference between each GDM subtype and NGT group. *P* values for pairwise comparisons were adjusted by Bonferroni correction. In order to eliminate impacts of confounds on difference among groups, we conducted linear or logistic regression models to adjust covariates. All the statistical analysis was performed by SPSS 22.0 (SPSS Inc., Chicago, IL). A two-sided *P *< 0.05 was considered statistically significant.

## Results

### Baseline characteristics

In these 456 pregnant women, two hundred and six women finally developed GDM. Further, 35 women were classified into GDM-resistance group. 43 women were eligible for the GDM-dysfunction group and 79 women, commodity with insulin resistance and beta cell dysfunction, were in the GDM-mix group. Baseline characteristics of the NGT (n = 250), GDM-resistance (n = 35), GDM-dysfunction (n = 43), GDM-mix (n = 79) and all GDM groups (n = 206) are presented in Table 1. Women in the GDM group were older than women with NGT (32 ± 5.0 vs 29 ± 5.0 years, *P *< 0.001) and prepregnancy and maternal BMI in the GDM group were higher than those in the NGT group (22.0 ± 3.7 vs 20.8 ± 3.7 kg/m^2^, *P *< 0.001; 23.2 ± 3.7 vs 21.8 ± 3.4 kg/m^2^, *P *< 0.001, respectively). Among different subtypes in GDM, women in the GDM-mix group were also older than women with NGT (32 ± 6.0 vs 29 ± 5.0 years, *P *< 0.001) and the levels of prepregnancy and maternal BMI were much higher compared to women in the NGT group (23.2 ± 4.0 vs 20.8 ± 3.7 kg/m^2^, *P *< 0.001; 24.5 ± 4.3 vs 21.8 ± 3.4 kg/m^2^, *P *< 0.001, respectively). Interestingly, women with GDM-resistance and women with NGT were similar in terms of age, prepregnancy and maternal BMI. Still, we observed similar levels of BMI change and the percentage of nulliparous between three subtype groups and NGT group.

### Glucose, insulin and indices of beta cell function and insulin resistance

**Fig. 1:**
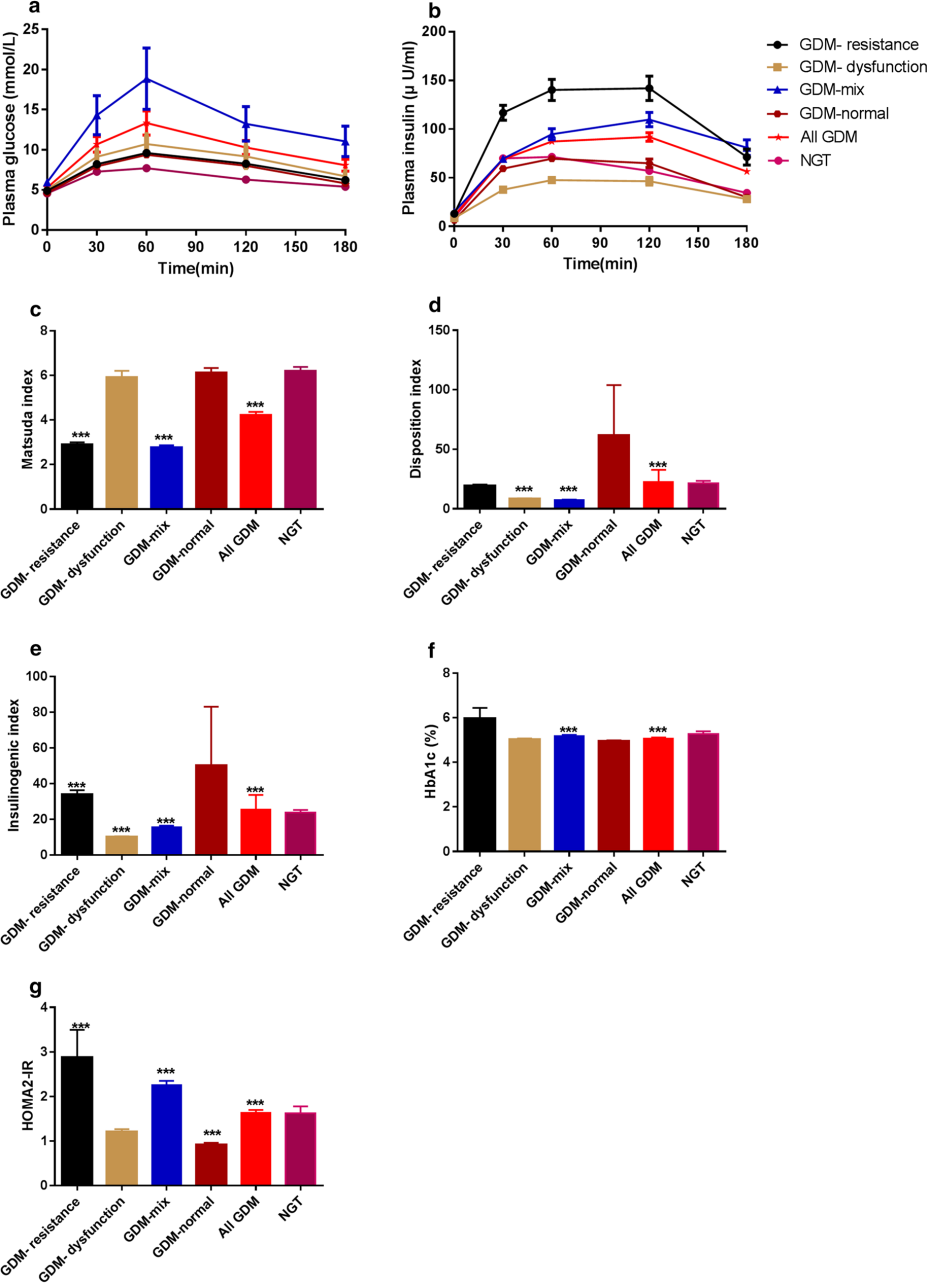
Dynamic responses of 3 h 75 g OGTT and glucose metabolism parameters among different GDM subgroups. Data are mean ± SEM. ***P < 0.001

Parameters of 75 g 3 h OGTT, measurements of beta cell function and insulin resistance and status of glucose metabolism (GA and HbA1c) were listed in Table 2 and the dynamic responses of glucose and insulin are portrayed in Fig. 1. Noticeably, levels of glucose on all the time points in the GDM-mix group were significantly higher than those in the NGT group (All *P *< 0.001), whereas the other two groups exhibited elevated levels of glucose on all the time points except for glucose 180 min compared to the NGT group. Moreover, three subgroups showed respective traits in insulin secretion patterns: levels of insulin in all the time points in the GDM-resistance group showed significant increment than those in the NGT group; women in GDM-dysfunction showed a lower beta cell responsiveness in early phase (30 min and 60 min) of insulin secretion according to the reference of women in the NGT group and levels of insulin on other time points showed no difference between these two groups; in addition, except of insulin 30 min, levels of insulin on all the other time points in the GDM-mix group were significantly higher than those in the NGT group.

**Table 2:** Comparison of glucose levels during 3 h 75 g OGTT and insulin secreting parameters among GDM subgroups and women with NGT

	GDM-resistance	*P* value	GDM-dysfunction	*P* value	GDM-mix	*P* value	All GDM	*P* value	NGT
Glucose 0 min (mmol/L)	4.8 (0.6)	0.017	5.1 (0.7)	< 0.001	5.2 (1.0)	< 0.001	5.0 (0.8)	< 0.001	4.6 (0.4)
Glucose 30 min (mmol/L)	8.3 (1.7)	0.008	8.2 (1.4)	< 0.001	9.0 (1.6)	< 0.001	8.5 (1.6)	< 0.001	7.3 (1.5)
Glucose 60 min (mmol/L)	9.8 (2.3)	< 0.001	10.1 (1.9)	< 0.001	10.6 (1.9)	< 0.001	10.2 (2.0)	< 0.001	10.2 (2.0)
Glucose 120 min (mmol/L)	8.6 (2.1)	< 0.001	8.0 (2.8)	< 0.001	9.2 (2.7)	< 0.001	8.6 (2.3)	< 0.001	7.7 (1.9)
Glucose 180 min (mmol/L)	6.1 (2.3)	0.069	6.2 (2.5)	> 0.999	7.5 (2.9)	< 0.001	6.5 (3.0)	< 0.001	6.4 (1.8)
Insulin 0 min (μU/mL)	11.9 (4.9)	< 0.001	7.6 (4.2)	0.628	13.6 (5.9)	< 0.001	10.4 (7.1)	< 0.001	8.3 (5.3)
Insulin 30 min (μU/mL)	106.9 (40.2)	< 0.001	37.0 (27.3)	< 0.001	64.8 (39.9)	> 0.999	59.4 (43.2)	0.356	62.9 (48.9)
Insulin 60 min (μU/mL)	122.0 (61.6)	< 0.001	46.0 (30.2)	0.001	86.2 (48.1)	< 0.001	76.8 (54.9)	0.006	65.0 (46.0)
Insulin 120 min (μU/mL)	126.7 (86.2)	< 0.001	47.9 (38.8)	> 0.999	95.9 (68.0)	< 0.001	77.2 (69.6)	< 0.001	49.3 (53.2)
Insulin 180 min (μU/mL)	63.0 (61.2)	0.003	22.3 (35.5)	> 0.999	67.0 (72.0)	< 0.001	41.5 (59.2)	< 0.001	27.3 (38.2)
HOMA2-IR	1.7 (0.7)	< 0.001	1.2 (0.9)	> 0.999	2.1 (1.1)	< 0.001	1.5 (1.1)	< 0.001	1.2 (0.8)
Matsuda index	2.9 (0.9)	< 0.001	5.4 (1.6)	> 0.999	2.8 (1.2)	< 0.001	3.7 (2.7)	< 0.001	5.6 (3.4)
Insulinogenic index	29.21 (18.5)	< 0.001	9.1 (5.5)	< 0.001	14.4 (9.5)	< 0.001	14.7 (11.5)	< 0.001	21.1 (14.5)
Disposition index	16.0 (9.1)	> 0.999	8.3 (3.4)	< 0.001	7.0 (4.6)	< 0.001	10.3 (8.4)	< 0.001	17.8 (12.1)
GA (%)	11.0 (1.7)	0.017	11.7 (1.9)	> 0.999	11.5 (2.0)	> 0.999	11.6 (2.0)	0.975	11.6 (2.1)
HbA1c (%)	5.2 (0.6)	0.125	5.0 (0.5)	> 0.999	5.2 (0.4)	< 0.001	5.1 (0.4)	< 0.001	5.0 (0.4)

Data are median (IQR) for continuous variables

Differences between GDM subgroups and women with NGT were evaluated with the Kruskal–Wallis test for continuous variables

When the *P* value was significant (< 0.05), Dunn’s multiple comparisons test was performed to evaluate the difference between each GDM subtype and NGT group. *P* values for pairwise comparisons were adjusted by Bonferroni correction

Reduced insulin sensitivity assessed by HOMA2-IR and Matsuda index was observed in the GDM-resistance group and the GDM-mix group compared to these in the NGT group. Furthermore, beta cell function adjusted for insulin resistance (HOMA2-IR)-disposition index showed a significant reduction in the GDM-dysfunction group and the GDM-mix group in comparison to the NGT group. Additionally, higher levels of GA and HbA1c were observed in the GDM-resistance group and the GDM-mix group, respectively (Table 2).

### Heterogeneity of perinatal outcomes in subtypes groups

Perinatal outcomes in subtypes groups were shown in Table 3. Compared with perinatal outcomes of women with NGT, women in GDM-mix group more likely to be subjected to LGA (*P *= 0.008) and adverse perinatal outcomes (*P *= 0.005). Nevertheless, these differences were normalized after adjusting age, prepregnancy and maternal BMI (GDM-mix vs. NGT: *P *= 0.186 for LGA and *P *= 0.141 for adverse outcomes) perinatal outcomes of women in the GDM-resistance and GDM-dysfunction group exhibited no difference with these of women with NGT. Interestingly, All the women diagnosed with GDM were more vulnerable to undergo cesarean delivery and one of adverse outcomes compared to women with NGT, whereas possibilities of other individual GDM-associated adverse outcomes exhibited no difference between them.

**Table 3:** Perinatal outcomes among groups with different glucose tolerance and insulin secreting status

	GDM-resistance	*P* value	GDM-dysfunction	*P* value	GDM-mix	*P* value	All GDM	*P* value	NGT
Gestational age (week)	39.7 (1.8)	–	39.4 (1.2)	–	39.3 (2.0)	–	39.4 (1.6)	0.964	39.5 (1.6)
Infant birth weight (g)	3260.0 (547.5)	–	3380.00 (575.0)	–	3380.0 (720.0)	–	3365.0 (527.5)	0.546	3340.0 (520.0)
Infant birth weight (z score)	0.20 (1.1)	–	0.36 (1.4)	–	0.41 (1.5)	–	0.31 (1.3)	0.186	0.14 (1.2)
BPD (cm)	93 (5.3)	–	93 (4.3)	–	93 (7.0)	–	93 (6.0)	0.149	94 (5.0)
FL (cm)	68 (3.5)	–	69 (4.3)	–	69 (4.0)	–	69 (4.0)	0.836	69 (4.0)
AFI (mm)	125 (49.8)	–	115 (32.0)	–	116 (41.0)	–	118 (43.75)	0.92	119 (38.5)
Apgar score	10 (0)	–	10 (0)	–	10 (0)	–	10 (0)	0.364	10 (0)
Infant male gender [n, (%)]	17 (48.6)	–	20 (46.5)	–	40 (50.6)	–	104 (50.5)	0.456	135 (54.0)
LGA [n, (%)]	2 (5.7)	0.4	6 (14.0)	0.808	20 (25.3)	*0.008*	34 (16.5)	0.263	32 (12.8)
Cesarean delivery [n, (%)]	17 (48.6)	–	19 (44.2)	–	40 (50.6)	–	95 (46.1)	0.029	90 (36.0)
Neonate hypoglycemia [n, (%)]	4 (11.4)	–	4 (9.3)	–	11 (13.9)	–	21 (10.2)	0.510	21 (8.4)
Any adverse outcome [n, (%)]	19 (54.3)	0.313	22 (51.2)	0.469	50 (63.3)	*0.005*	114 (55.3)	0.031	113 (45.2)

Data are median (IQR) for continuous variables and n (%) for categorical variables

Differences between GDM subgroups and women with NGT were evaluated with the Kruskal–Wallis test for continuous variables and χ^2^ or Fisher exact test for categorical variables

When the P value was significant (< 0.05), Dunn’s multiple comparisons test or χ^2^ or Fisher exact test was performed to evaluate the difference between each GDM subtype and NGT group. *P* values for pairwise comparisons were adjusted by Bonferroni correction

## Discussion

Here, we conducted a prospective cohort study in a relatively large number of women with gestational diabetes and analyzed perinatal outcomes according to the heterogeneity of insulin resistance and beta cell dysfunction-a novel perspective about GDM. Three phenotypic subgroups were defined: GDM-resistance group, GDM-dysfunction group and GDM-mix group. Then women in the GDM-resistance and GDM-dysfunction was further characterized by a predominant insulin resistance (Matsuda index, OGTT) or a decreased beta cell function in response to oral glucose (disposition index, OGTT) and women in the GDM-mix group hold the combination of features about these two groups, so the most divergent metabolic disorder was observed in the GDM-mix group compared with women in the NGT group, but other two groups. Not surprisingly, the incremental risk of GDM-related complications and LGA were observed in the GDM-mix group compared to women in the NGT group, although this difference was attenuated for the adjustment of confounding factors including age, prepregnancy and maternal BMI.

One strength of our study is that we performed a clinical investigation with detailed responses of glucose and insulin to OGTT and the association between pathophysiology of GDM and perinatal outcomes in a prospective cohort of pregnant women without prior diabetes history. Five individual time points (0 min, 30 min, 60 min, 120 min and 180 min) measurements of glucose and insulin outlined a detailed and complete picture of glucose and insulin fluctuations during OGTT, which provided clear and exact information about insulin resistance and beta cell function on different phenotypes about GDM. Moreover, the level of prepregnancy BMI was documented and it played, to some extent, an index in estimating the insulin sensitivity before pregnancy.

Given similar genetic background and pathophysiological mechanism [23, 24], GDM should be considered as a heterogeneous disease, like T2D. We used indices evaluating insulin resistance and beta cell function to investigate whether their heterogeneity contributes to GDM-related complications, though many researches applied other parameters, such as BMI or glucose [5, 12–15], as the reference. Recently, Powe et al. [3] also investigated the relationship between insulin resistance and beta cell function and perinatal outcomes in women with GDM. Different from our discoveries, they found that women with GDM undergoing impaired insulin sensitivity were more likely to confer GDM-related complications, independent from maternal BMI. Factors that may have contributed to this difference include: firstly, they took the advantage of the Stumvoll first-phase estimate to exhibit beta cell function, not DI, a more widely used index and adjusted for insulin resistance [17]; second, we conducted a complete Chinese women cohort, therefore, the divergent ethnological genetic and environmental background induced a disparate metabolic pattern on GDM: as we can see, levels of fasting insulin were much higher in the GDM-resistance and GDM-mix group than these in the NGT group, however, this observation was completely opposite from Powe’s investigation. In that, we are inclined to draw this explanation that ethnological differences prompted separate a metabolic pattern and disorder in our cohorts and it may leaded to differences on perinatal outcomes between these two researches. Indeed, the number of subjects in our cohorts was much smaller than Powe’s investigation and it may undermine our conclusion and confounded our observation. However, considering the collective role of insulin resistance and beta cell dysfunction in promoting the occurrence of GDM [23, 24], it is reasonable to draw the conclusion that women in the GDM-mix group hold most severe metabolic disorder and further greatest risk of GDM-related complications among three subtypes.

A limitation of our investigation is unavailable of assessing levels of glucose and insulin before, after and during the pregnancy, and these longitude transformations of insulin resistance and beta cell function facilitate us to portrait a more clear picture about impacts of heterogeneous insulin resistance and beta cell dysfunction on prenatal outcomes. Moreover, the limited sample size and monotonous ethnological subjects may partly diminish our conclusion. Furthermore, the lack of data about levels of insulin in infants hampers further analyses and evaluations on impacts of different subtypes on the neonate hyperinsulinemia, or the association between hyperinsulinemia and birthweight or neonate hypoglycemia. Finally, our study did not include genetic information and adipokines associated with insulin resistance and beta cell function.

## Conclusion

In general, our study revealed that women with GDM display respective characteristics on metabolism disorders and confer discriminating risk of adverse perinatal outcomes in accordance with various phenotypes of insulin resistance and beta cell function. Among three subtypes of GDM, women with the combination of predominant insulin resistance and beta cell dysfunction conferred the greatest risk of adverse perinatal outcomes. Currently, few studies concentrated on the heterogeneity of GDM and its influences on perinatal outcomes, especially on the base of pathogenesis of GDM. Future researches, including large number of subjects and diverse races, may help to explain the underlying causal and effect link between subtypes in pathogenesis of GDM and perinatal outcomes.

## Additional file

**Additional file 1: Table S1.** Perinatal Outcomes of Women with GDM Who were not taken into subgroup analyses.

## Abbreviations

GDM: gestational diabetes mellitus
T2D: type 2 diabetes
LGA: large for gestational age
OGTT: oral glucose tolerance test
IADPSG: International Association of Diabetes and Pregnancy Study Group
BMI: body mass index
NGT: normal glucose tolerance
GDM-dysfunction: GDM with a predominant beta cell dysfunction
GDM-resistance: GDM with a predominant insulin resistance defect
GDM-mixed: GDM with both traits mentioned above
GA: glycated serum albumin
GDM-normal: GDM without insulin resistance defect and beta cell dsyfunction
