# Supplementary material for: Heterogeneity of insulin resistance and beta cell dysfunction in gestational diabetes mellitus: a prospective cohort study of perinatal outcomes
Source: J Transl Med. 2018 Oct 24;16:289. doi: 10.1186/s12967-018-1666-5 (PMC6201515; doi:10.1186/s12967-018-1666-5)
Supplement: Supplementary file 1 — Additional file 1: Table S1. Perinatal Outcomes of Women with GDM Who were not taken into subgroup analyses. [file 12967_2018_1666_MOESM1_ESM.doc]

**Table S1. Perinatal Outcomes of Women with GDM Who were not taken into subgroup analyses**

|  | **GDM-normal** | ***P* value** | **NGT** |
| --- | --- | --- | --- |
|  |  |  |  |
| **Gestational age (week)** | 39.4 (1.5) | 0.837 | 39.5 (1.6) |
| **Infant birth weight (g)** | 3350.0(505.0) | 0.939 | 3340.0(520.0) |
| **Infant birth weight (z score)** | 0.15 (1.2) | 0.685 | 0.14 (1.2) |
| **BPD (cm)** | 93 (5.0) | 0.623 | 94 (5.0) |
| **FL (cm)** | 70 (3.0) | 0.192 | 69 (4.0) |
| **AFI (mm)** | 119 (48.5) | 0.853 | 119 (38.5) |
| **Apgar score** | 10 (0) | 0.658 | 10 (0) |
| **Infant male gender [n,(%)]** | 27 (55.1) | 0.878 | 135 (54.0) |
| **LGA [n,(%)]** | 7 (13.7) | 0.879 | 32 (12.8) |
| **Cesarean delivery [n,(%)]** | 19 (38.8) | 0.712 | 90 (36.0) |
| **Neonate hypoglycemia [n,(%)]** | 2 (4.1) | 0.392 | 21 (8.4) |
| **Any adverse outcome [n,(%)]** | 23 (46.9) | 0.878 | 113 (45.2) |

Data are median (IQR) for continuous variables and n (%) for categorical variables

Differences between GDM who were not taken into subgroup analyses and women with NGT were evaluated with the Mann-Whitney U test for continuous variables and χ^2^ or Fisher exact test for categorical variables.
